# Infection prevention and patient safety improvement in developing countries thanks to sodium hypochlorite production devices

**DOI:** 10.1186/2047-2994-4-S1-I8

**Published:** 2015-06-16

**Authors:** D von der Weid

**Affiliations:** 1 Fondation Antenna Technologies, Switzerland

## Introduction

The technology developed by Antenna Technologies has allowed reaching some promising results. In Guinea Conakry, The sales of Sodium Hypochlorite (SH) flasks in a limited zone (target population for 2014: 7'218’882) represent a calculated coverage ratio of 27,5% (2014: 3'390'637 flasks sold at very low price to lowest income group). In Burkina Faso, the excellent results obtained by a pilot study under the auspices of the Health Ministry suggests that a scaling-up phase with the ambition to equip 63 health districts and 9 regional hospitals should be undertaken in a close future.

## Objectives

To demonstrate that devices, through the electrolysis of saltwater, can locally produce high-standard quality SH at a very low cost and without prior scientific knowledge. This SH allows to significantly improve public health and patient safety (disinfection) particularly in developing countries.

## Methods

Antenna Technologies (AT) assesses, through experiences and data from the field, the usefulness of its devices (WATA) and chemical reagents (WataTest, WataBlue) by considering growing demand for its technology and its demonstrated substitutive capacity.

## Results

AT’s devices are now used in thirty countries. Laboratory empirical studies and data gathered on the field have shown that such devices are able to produce on a regular basis a SH equivalent to 6g/L of active chlorine. This concentration is in line with recommendations of CDC for the disinfection in health centers in the Ebola context (CDC, 2015) and with the recommendations of the WHO regarding infection control in health care facilities (WHO, 2004). HS is also recognized as an efficient disinfectant for drinkable water. Water quality is expected to have a huge influence on the prevalence of waterborne diseases. Chlorination is identified as the most cost-effective disinfection solution.

## Conclusion

SH production process is very low-cost and easy to learn/train. The produced SH meets high-quality standards. According to CDC and WHO, the SH is an efficient and convenient disinfectant/antiseptic for health facilities and water, especially in developing countries. SH is also relevant for the washing and disinfection of the wounds (Dakin solution).

## Disclosure of interest

None declared.

